# Poly-*ε*-Caprolactone Microsphere Polymers Containing Usnic Acid: Acute Toxicity and Anti-Inflammatory Activity

**DOI:** 10.1155/2017/7392891

**Published:** 2017-12-04

**Authors:** Jéssica A. P. Barbosa, Eryvelton S. Franco, Camilla V. N. S. Silva, Tatiane O. Bezerra, Marllon A. N. Santana, Carlson H. R. C. Júnior, Teresinha G. Silva, Noemia P. S. Santos, Maria B. S. Maia

**Affiliations:** ^1^Department of Antibiotics, Federal University of Pernambuco, Recife, PE, Brazil; ^2^Department of Physiology and Pharmacology, Federal University of Pernambuco, Recife, PE, Brazil; ^3^Department of Pharmaceutical Sciences, Federal University of Pernambuco, Recife, PE, Brazil; ^4^Laboratory of Biotechnology and Pharmaceuticals, Academic Center of Vitória, Federal University of Pernambuco, Vitória de Santo Antão, PE, Brazil

## Abstract

Usnic acid (UA) has been studied by its pharmacological properties; however, it presents moderate toxicity, low solubility, and absorption by biological membranes. The aim of this study was to develop poly-*ε*-caprolactone microsphere polymers containing UA (UA-micro) and evaluate their acute toxicity and anti-inflammatory activity. The microspheres were prepared by multiple emulsion technique (water/oil/water) and characterized by the encapsulation efficiency, particle size, polydispersity index, and zeta potential. The acute toxicity of UA and UA-micro (25–50 mg/kg; p.o.) was evaluated in mice. The anti-inflammatory activity of UA and UA-micro was evaluated by subcutaneous air pouch and carrageenan-induced paw edema in rat, with measurement of inflammatory cytokines and MPO levels. The UA presented encapsulation efficiency of 97.72%, particle size of 13.54 micrometers, polydispersity index of 2.36, and zeta potential of 44.5 ± 2.95 mV. The UA-micro presented lower acute toxicity (LD_50_ value up to 2000 mg/kg; p.o.) when compared to UA. UA-micro and UA (25 mg/kg) significantly reduced paw volume and decreased MPO levels, whereas only UA-micro (50 mg/kg) reduced significantly IL-1*β*, TNF-*α*, and NO levels in inflammatory exudate. These results suggest that controlled release systems, as microspheres, can be a promising alternative to reduce the toxicity of UA, making it a viable compound for inflammation therapy.

## 1. Introduction

Inflammation is a complex process involving several cell types and molecular mediators, whose main goals are to isolate, neutralize, and destroy harmful agents, following tissue regeneration stimuli [1]. Although nonsteroidal anti-inflammatory use in therapeutics has its efficacy, prolonged treatment may be responsible for gastrointestinal side effects, such as superficial mucous membrane alterations, erosive gastritis, and peptic ulcer related to prostaglandins inhibition, produced via COX-1 [2]. There is a growing search for new anti-inflammatory agents from natural products, mainly for compounds that have therapeutic and pharmacological potential, reduced side effects, and low cost [3].

Usnic acid (2,6-diacetyl-7,9-dihydroxy-8,9b-diethyl-1,3(2H,9bH)-benzofuran, C18H16O7), a secondary metabolite isolated from lichens [4, 5], has been highlighted for presenting several biological activities, such as anti-inflammatory, analgesic [6], antimicrobial [7], gastroprotective, antioxidant [8], anticancer [9], and tissue healing activities [10]. Although usnic acid has a great variety of biological activities, its utilization as therapeutic agent is not possible yet due to its physicochemical properties, such as low solubility, high toxicity, and impaired interaction with cell barriers [11, 12]. Issues regarding usnic acid administration can be overcome through a controlled-release drug system that can reduce usnic acid toxicity and increase therapeutic efficacy.

The controlled-release drug system was designed to slowly and continually release the drug, keeping the desired concentration in the blood for a longer period. One of the controlled-release drug systems is microspheres, which are polymeric solid particles (usually spherical) that deserve highlight due to physicochemical and biological stabilities. In addition, microspheres are utilized for encapsulation of several substances, such as peptides, proteins, DNA/RNA, and drugs [13, 14]. Encapsulation of usnic acid into microspheres may overcome boundaries regarding low solubility and difficult absorption through cell membranes to reduce the toxic effects caused by free usnic acid, which may increase its biological effect.

## 2. Materials and Methods

### 2.1. Animals

Male Wistar rats (190–200 g) and female Swiss mice (30–35 g) from the vivarium of Antibiotics Department, Federal University of Pernambuco (UFPE), were used. Animals were kept in polypropylene boxes in a room with controlled temperature (22 ± 2°C) and light-dark cycle of 12 h/12 h. Water and food were available ad libitum. Experimental protocols were approved by the Animal Studies Committee (CEEA) of the UFPE, under process number 23076.052269/2012-35.

### 2.2. Reagents

All the reagents (poly-*ε*-caprolactone, polyvinyl alcohol (MW: 30.000–70.000), polyethylene glycol (MW: 3.350), usnic acid, trehalose, indomethacin, and carrageenan type IV) were obtained from Sigma-Aldrich (St. Louis, USA).

### 2.3. Preparation of PCL Microspheres Containing Usnic Acid

Microspheres were prepared using multiple emulsion method following solvent evaporation [15]. One simple emulsion was prepared by dissolving 225 mg of poly-*ε*-caprolactone and 25 mg of usnic acid into dichloromethane (6 mL). The organic solution was emulsified (Ultra-Turrax T-25, IKA) with 125 mg of polyethylene glycol into water for 1 min at 6848 ×g. The produced emulsion was added to an aqueous phase constituted by polyvinyl alcohol (1.25%) and was emulsified during 30 s at 6848 ×g, resulting in multiple emulsions. The final emulsion was kept under agitation at 17 ×g for 2 hours. Microspheres were centrifuged at 963 ×g for 10 minutes and dispersed into trehalose 1% (m/v). Subsequently, microspheres were frozen at −80°C and lyophilized afterwards.

### 2.4. Physicochemical Characterization of Poly-*ε*-Caprolactone Microspheres Containing Usnic Acid

Usnic acid encapsulation efficiency in the microspheres was determined by spectrophotometry (Ultrospec 3000, Amersham Pharmacia Biotech, USA). Lyophilized microsphere samples (5 mg) were diluted in a dichloromethane : methanol (3 : 2) mixture and submitted to sonication for 10 min. An aliquot of this solution was diluted to a theoretical concentration of 6 *μ*g·mL^−1^. Usnic acid was determined by spectrophotometry (280 nm) using a standard curve of 3–10 *μ*g·mL^−1^ in acetonitrile.

Microsphere particles size was determined through laser diffraction technique using a particle analyzer (Zetasizer Nano ZS90, Malvern, UK) [16]. Lyophilized microsphere samples (10 mg) were dispersed into a Tween® 80 aqueous solution at 0.04% (w/v) and submitted to sonication for 10 min. Calculated parameters were as follows: volume median diameter (VMD) and span index, calculated by span equation = (*d*(*v*; 0.9) −* d*(*v*; 0.1))/*d*(*v*; 0.5), where* d*(*v*; 0.9),* d*(*v*; 0.1), and* d*(*v*; 0.5), represent the percentage of microspheres with volume median diameter above 90%, 10%, and 50%, respectively.

Surface charge (zeta potential) was determined by electrophoretic mobility method using the Zetasizer Nano ZS90 (Malvern, UK) equipment and Zetasizer software. Microsphere zeta potential (*ζ*) was measured after dilution into an aqueous solution of 1 Mm NaCl (0.5%, p/v) at 25°C.

### 2.5. Acute Toxicity

Acute toxicity tests were designed following the “Acute Toxic Class Method” [17]. Mice were divided into five groups (*n* = 3 animals/group). Experiments were repeated using the same animal quantity, with a total of 6 animals/group. The control group received vehicle (0.9% NaCl: 5% Cremophor; p.o.) and experimental groups received usnic acid (UA, 2000, 300, and 50 mg/kg; p.o.), poly-*ε*-caprolactone microsphere polymers containing usnic acid (UA-micro, 2000 mg/kg; p.o.), or indomethacin (10 mg/kg; p.o.). Animals were observed during the first 8 and 24 hours during 14 days after compound administration and had toxicity signals scored. On the last day of observation, animals were euthanized in a CO_2_ chamber and had their organs (liver and kidney) removed for histopathological analysis.

### 2.6. Histopathological Analysis

Necropsy of animals submitted to acute toxicity was performed and the organs (liver and kidney) were kept in neutral buffered formalin (10%) for 48 h at room temperature. Then, organs were submitted to dehydration using ethyl alcohol in different concentrations, diaphanized in xylol, impregnated, and included in paraffin. Samples were sliced into a microtome adjusted to 5 *μ*m. Histological slices were kept in an incubator at 37°C for 24 h in order to dry. Subsequently, slices were tinted with hematoxylin-eosin stain (H&E). Analysis was made using optical microscope.

### 2.7. Carrageenan-Induced Paw Edema

Paw edema test was performed according to Winter et al. [18]. Five Wistar rat groups (*n* = 5 animals/group) were treated orally with UA (25 mg/kg), UA-micro (25 and 50 mg/kg), indomethacin (10 mg/kg), or vehicle. One hour after treatments, 0.1 ml of a commercially prepared carrageenan was inoculated into the right hind of animals. The volume of the hind paw swelling (ml) was measured with an electronic water plethysmometer (Ugo Basile, Italy) at zero minutes (before) and 1, 2, 3, and 4 h after a carrageenan injection.

### 2.8. Myeloperoxidase (MPO) Levels Quantification

Paw tissues of animals submitted to paw edema test were collected 6 hours after carrageenan administration. Samples were weighted, ground, and kept in phosphate buffer [50 mM, pH 6.0, and 0.5% hexadecyltrimethylammonium bromide (HTAB, Sigma Chem. Co, USA)]. Samples were centrifuged at 12.000 ×g for 2 minutes at 4°C. Each well in a 96-well plate was filled with 5 *μ*L of centrifuged supernatant and 200 *μ*L of o-dianisidine dihydrochloride solution (Sigma Chem. Co, USA; 0,167 mg/mL, prepared in 50 mM potassium phosphate buffer with 0.005% H_2_O_2_) following spectrophotometer reading (450 nm). Results were expressed in MPO units (UMPO/mg of tissue), considering that 1 UMPO corresponds to the quantity that one enzyme degrades with 1 *μ*moL per minute [19]. The test was performed in duplicates.

### 2.9. Subcutaneous Air Pouch (SAP) Test

Anti-inflammatory activity was evaluated through air pouch formation by injection of 2.5 mL of sterile air on day 0 in the dorsal region of the mice, followed by a second injection of 2.5 mL of sterile air three days after the first. On the sixth day, according to their group, the animals received orally vehicle, UA (25 mg/kg; p.o.), UA-micro (25 and 50 mg/kg), or indomethacin (10 mg/kg). One hour after treatments, inflammation was induced by injection of 1 mL carrageenan (1%) inside the air pouch. After 6 h, animals were euthanized in a CO_2_ chamber and had the pouch washed with 3 mL of PBS containing 3 *μ*Mol EDTA. Polymorphonuclear leukocytes quantification on the lavage fluid was performed using an ABX Micros 60 hematology analyzer [20, 21].

### 2.10. Nitric Oxide (NO) Quantification

To access nitric oxide (NO) production, nitrate concentrations (NO stable metabolite) were quantified in the air pouch exudate through air pouch test. One sample aliquot of 50 *μ*L was transferred into a microplate and incubated with 50 *μ*L Griess reagent (sulfanilamide 1%, naphthyl ethylenediamine dichlorohydrate 0.1%, and H_3_PO_4_ 5%) for 10 min at room temperature in the dark. Absorbance (540 nm) was obtained using a microplate reader and nitrate concentration was calculated using a sodium nitrate standard curve [22].

### 2.11. TNF-*α* and IL-1*β* Quantification

TNF-*α* and IL-1*β* quantification was accessed using specific ELISA kits for mice, following manufacturer's instructions (BD Biosciences, San Diego, California, USA). The lower limit of detection was 10 pg/mL.

### 2.12. Statistical Analysis

Results were expressed as mean ± standard deviation for each experimental group using GraphPad Prism software (version 6.0; DEMO). Data were analyzed through one-way Analysis of Variance (ANOVA) followed by Tukey's test, except for paw edema test, which was analyzed through two-way Analysis of Variance (ANOVA) followed by Bonferroni multiple comparison test. Confidence intervals of 95% and “*p*” values lower than 0.05 (*p* < 0.05) were considered as indicators of statistical significance.

## 3. Results

### 3.1. Physicochemical Characterization of Poly-*ε*-Caprolactone (PCL) Microspheres Containing Usnic Acid

Encapsulation efficiency was obtained through three analytic curves, which were prepared from different stock solutions, and adjusted through linear regression analysis. The equation mean that was obtained from three calibration curves was *y* = 0.07309*x* + 0.00263, where *y* is the absorbance (nm) and *x* is the concentration (*μ*g/mL) in usnic acid equivalents. The correlation coefficient was 0.99989, meaning that 99.98% of the total variation around the mean was explained by linear regression. Usnic acid was encapsulated into PCL microspheres with efficiency of 97.72 ± 0.0004%. UA-micro zeta potential (*ζ*) had a higher negative value (*ζ* = −44.5 ± 2.95 mV) when compared to PCL microspheres (*ζ* = −26.9 ± 0.58 mV) (Table 1).

In this study, the grain size of microspheres with usnic acid was also verified, which was 13.54 *μ*m, while microspheres without usnic acid size had grain size of 9.37 *μ*m (Table 1). Microspheres without usnic acid (PCL microspheres) showed polydispersion index (span) of 2.18, while the UA-micro span was 2.36, which suggests homogenous formulations.

### 3.2. Acute Toxicity

Acute toxicity grade and physiological parameters (water and food intake, weight gain, and mortality) are presented in Table 2. The animals of UA-treated group (2000 mg/kg; p.o.) presented clinical symptoms of toxicity (irritability, vocal fremitus, and abdominal contortion) in the first 2 hours of observation, followed by death. Regarding the group treated with UA (300 mg/kg; p.o.), reduced food intake and weight loss were observed when compared to the control group. On the other hand, no indication of acute toxicity or occurrence of death was observed in the groups treated with UA-micro (2000 mg/kg; p.o.) or UA (50 mg/kg; p.o.) as compared to the control group.

### 3.3. Histopathological Analysis

**Figure 1:**
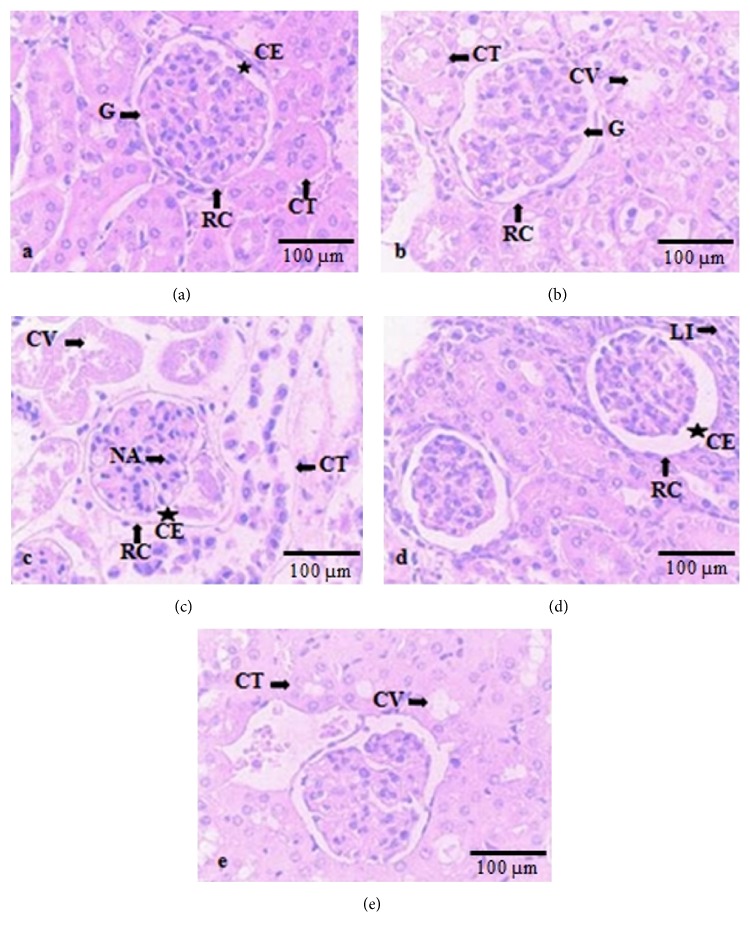
Kidney sections of (a) control, (b) UA-micro (2000 mg/kg; p.o.), (c) UA (2000 mg/kg; p.o.), (d) UA (300 mg/kg; p.o.), and (e) UA (50 mg/kg; p.o.). RC: renal corpuscle; CS: capsular space; LI: leukocytes infiltrate; CT: convoluted tubules; G: glomerulus; CV: cytoplasmic vacuolation; NA: nuclear acidophilia (×400 magnification).

In control animals, no morphological kidney tissue damage was detected (Figure 1(a)). The UA-micro-treated group (2000 mg/kg; p.o.) showed slight derangement of the simple cuboidal epithelium of convoluted tubules and vacuolation presence, while renal corpuscles were kept preserved (Figure 1(b)). On the other hand, as compared to control group, the UA-treated animals (2000 mg/kg; p.o.) exhibited serious kidney damage, indicated by intense nuclear acidophilia and cytoplasmic vacuolation, capsular space and convoluted tubules lumen size increase, fluid accumulation between the basal membrane and cuboidal epithelial cells of the convoluted tubes, and cytoplasmic material accumulation in the renal tubules and cell lysis (Figure 1(c)). Morphological changes with some damage in kidney tissue were observed in animals treated with UA at dose of 300 mg/kg (p.o.) (increasing capsular space and leukocyte infiltration) (Figure 1(d)) or 50 mg/kg (p.o.) (renal tubules nuclei number increased, showing cytoplasmic vacuolation (Figure 1(e)).

**Figure 2:**
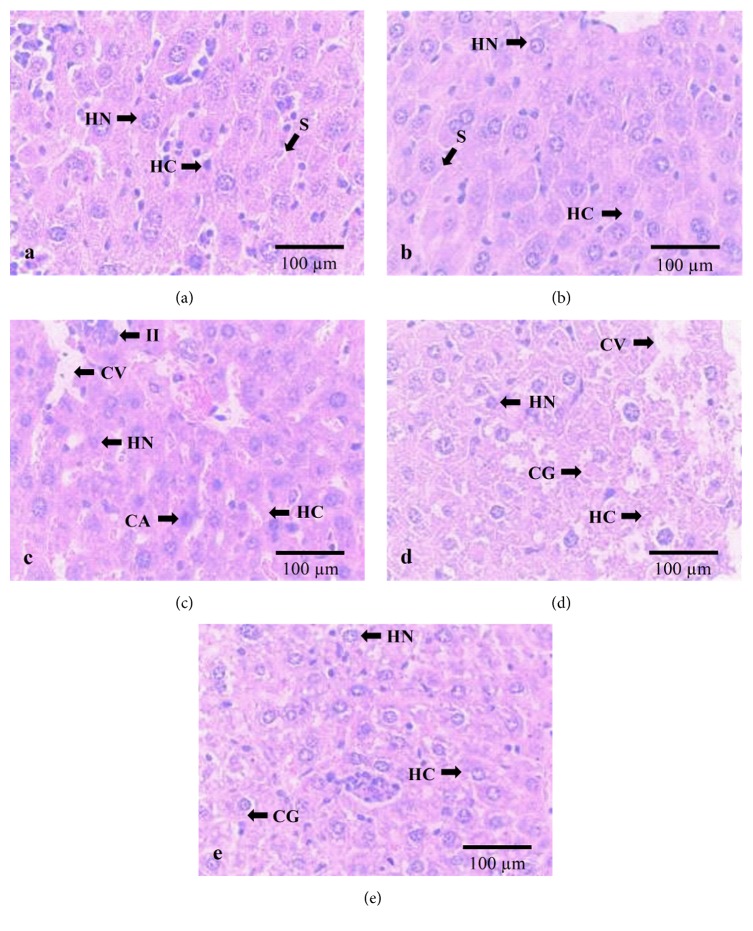
Liver sections of (a) control, (b) UA-micro (2000 mg/kg; p.o.), (c) UA (2000 mg/kg; p.o.), (d) UA (300 mg/kg; p.o.), and (e) UA (50 mg/kg; p.o.). S: hepatic sinusoids; HN: hepatocyte nucleus; HC: hepatic cell plates; CV: cytoplasmic vacuolation; II: inflammatory influx; CA: cytoplasmic acidophilia; CG: cytoplasmic granules (×400 magnification).

Concerning hepatic tissue, no abnormalities were observed in control group (Figure 2(a)). The UA-micro-treated animals (2000 mg/kg p.o.) showed plates of hepatic cells and sinusoids but preserved hepatocytes nuclei (Figure 2(b)). However, in mice treated with UA (2000 mg/kg; p.o.), hepatic cell plates derangement, intense vacuolation, inflammatory influx, cytoplasmic acidophilia, and decharacterization of the nucleus of hepatocytes were observed (Figure 2(c)). In UA-treated group (300 mg/kg), granule cytoplasmic accumulation, cytoplasmic vacuolation, hepatic cell plates derangement, and slight decharacterization of the hepatocyte nucleus (Figure 2(d)) were observed. In UA-treated animals (50 mg/kg; p.o.), hepatic cell plates were preserved, such as the hepatocyte nuclei, while almost no cytoplasmic granules were preserved (Figure 2(e)).

### 3.4. Carrageenan-Induced Paw Edema

Table 3 shows the evolution of paw edema in control and experimental groups. The rat paw edema volume in UA- (25 mg/kg; p.o.), UA-micro- (25 and 50 mg/kg; p.o.) or indomethacin- (10 mg/kg; p.o.) treated animals differs markedly from that observed in the corresponding control animals (Figure 1). As can be observed, the anti-inflammatory activity of free usnic acid (UA) was preserved when it was encapsulated in microspheres (UA-micro). In addition, this activity was dose-dependent and comparable to that presented by indomethacin (10 mg/kg; p.o.) in the time points of 1 and 2 hours.

### 3.5. Myeloperoxidase (MPO) Quantification

**Figure 3:**
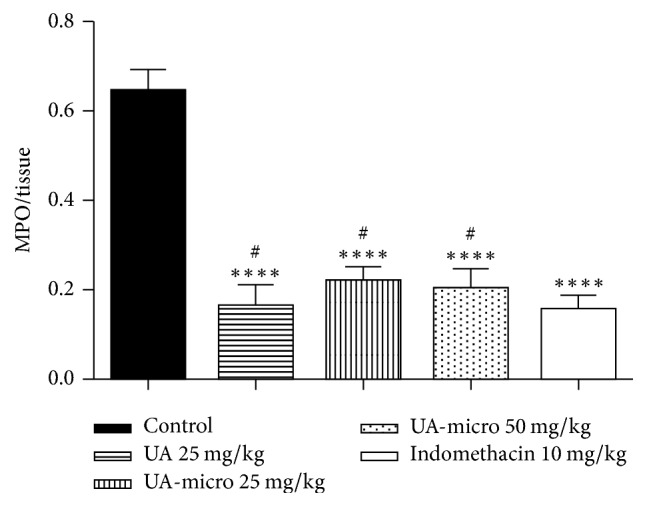
Effect of free usnic acid (UA), encapsulated usnic acid microspheres (UA-micro), or indomethacin on myeloperoxidase (MPO) levels in the subplantar tissue of rats paw. Values were expressed as mean ± SD. ^*∗∗∗∗*^*p* < 0.0001. ^#^Nonsignificant versus indomethacin.

As can be seen in Figure 3, baseline MO levels were significantly different in experimental and control groups. On the other hand, no significant differences among UA (25 mg/kg; p.o.), UA-micro (25 and 50 mg/kg; p.o.), or indomethacin (10 mg/kg; p.o.) were observed regarding MO levels (Figure 3).

### 3.6. Polymorphonuclear Leukocytes Migration

The treatment with UA (25 mg/kg; p.o.), UA-micro (25 and 50 mg/kg; p.o.), or indomethacin (10 mg/kg; p.o.) significantly reduced leukocytes migration to injured paws (Table 4). The neutrophil count was correlated with myeloperoxidase only in the CKD group.

### 3.7. TNF-*α*, IL-1*β*, and Nitric Oxide (NO) Levels

**Figure 4:**
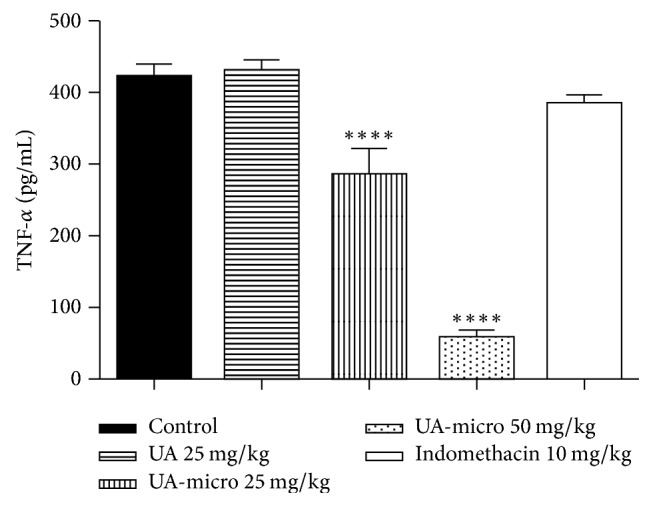
Effect of free usnic acid (UA), encapsulated usnic acid microspheres (UA-micro), or indomethacin regarding TNF-*α* concentration in the inflammatory exudate from subcutaneous air pouch test. Values were expressed as mean ± SD, and statistical significance between groups was determined using Analysis of Variance (ANOVA) followed by Tukey's test. ^*∗∗∗∗*^*p* < 0.0001.

**Figure 5:**
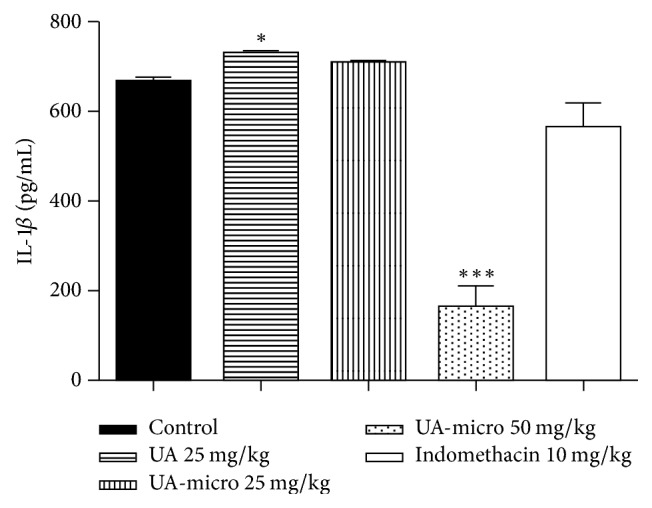
Effect of free usnic acid (UA), encapsulated usnic acid microspheres (UA-micro), or indomethacin on IL-1*β* concentration in the inflammatory exudate from subcutaneous air pouch test. Values were expressed as mean ± SD, and statistical significance between groups was determined using Analysis of Variance (ANOVA) followed by Tukey's test. ^*∗*^*p* < 0.01; ^*∗∗∗*^*p* < 0.001.

**Figure 6:**
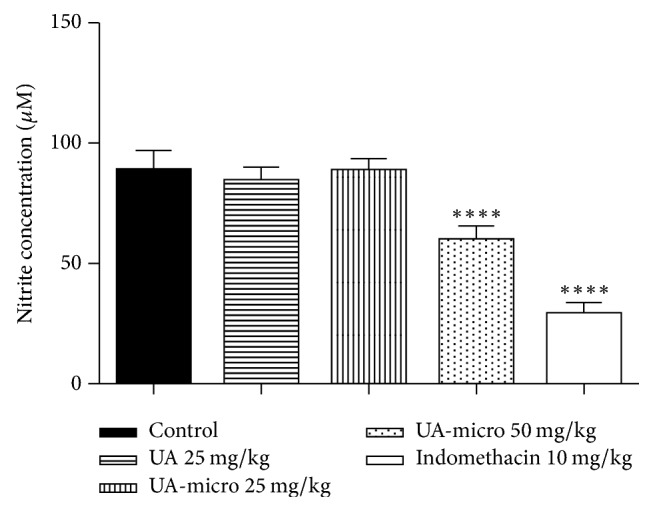
Effect of free usnic acid (UA), encapsulated usnic acid microspheres (UA-micro), or indomethacin regarding NO (*μ*M) concentration in the inflammatory exudate from subcutaneous air pouch test. Values were expressed as mean ± SD, and statistical significance between groups was determined using Analysis of Variance (ANOVA), followed by Tukey's test. ^*∗∗∗∗*^*p* < 0.0001.

Significant reductions were also observed in TNF-*α* (Figure 4), IL-1*β* (Figure 5), and NO (Figure 6) levels. In a dose-dependent manner, UA-micro inhibited the release of these chemical mediators from inflammation, whereas indomethacin (10 mg/kg; p.o.) reduced NO levels.

## 4. Discussion

Usnic acid, a secondary metabolite of lichen, is of great interest in pharmacological research. However, this compound has been associated with severe liver damage (hepatotoxicity) when taken as a dietary supplement. The anti-inflammatory activity of usnic acid has been described in the literature by several researchers using in vitro and in vivo assays [6, 23] (Su et al., 2014). In this context, our work aimed to compare the anti-inflammatory activity and acute toxicity of UA incorporated in poly-*ε*-caprolactone (PLC) microspheres (UA-micro) and free UA.

In relation to microsphere physicochemical characterization, our results showed that UA encapsulation into microparticles was satisfactory and suggest its further use for UA delivery application. Other drugs with similar hydrophobic characteristics to UA, such as paclitaxel [24], ibuprofen [25], and transdehydrocrotonin [11], and UA itself [26] showed high encapsulation efficiency into microspheres. Our results also showed that UA incorporated into PCL matrix microspheres increased microparticles size. Thus, it is possible to suggest that particles are homogeneous once polydispersion values (span) vary from 1.1 to 3.3. These data allow us to classify size distribution as monomodal, which is acceptable for controlled-release oral system [26]. Zeta potential can be used to determine microparticles surface charge and indicate their stability when in suspension. Moreover, their negative charge could be associated with the presence of drug molecules adsorbed on the microsphere polymer wall, which can influence surface charge [14].

Consistently, as incorporated into poly-*ε*-caprolactone microsphere polymers (UA-micro), UA showed reduced toxicity degree and maintained its anti-inflammatory activity. The downregulated acute toxicity verified in UA-micro (LD_50_ value up to 2000 mg/kg; p.o.) can be explained by its incorporation into microspheres. According to Chen et al. [27], poly-*ε*-caprolactone microparticles act as a drug delivery system, improving the therapeutic effect, prolonging the biological activity, controlling the drug release rate, decreasing the administration frequency, and so forth. These drug delivery systems protect certain labile active principles from degradation and/or inactivation by gastric juice, improve bioavailability by increasing the cellular penetration of substances, and provide release of the drug at the desired site of action, decreasing the toxic effects that normally accompany conventional therapy [28]. Regarding UA acute toxicity, our results corroborate with those found by Navarro et al. [29] which registered 100% death in mice treated with UA (2000 mg/kg; p.o.). Examination of liver and kidney tissue of animals treated with UA showed significant histopathological changes. Important liver architectural alterations (hepatic cell plates derangement, intense vacuolation, inflammatory influx, cytoplasmic acidophilia, and decharacterization of the nucleus of hepatocytes) were observed in mice treated with UA (2000 mg/kg; p.o.), whereas at the same dose UA-micro-treated animals showed plates of hepatic cells and sinusoids but preserved hepatocytes nuclei. Pramyothin et al. [30] found that UA acts altering cell membrane integrity, allowing for hepatic-specific enzymes release, mainly transaminases, causing mitochondrial function impairment, which culminates in cellular respiration control loss. In USA, hepatocellular damage was demonstrated when subjects ingested LipoKinetix®, a dietary supplement that contains UA [31]. According to Han et al. [32], this kind of damage happens due to the increase in reactive oxygen species production through the electron transport chain, which leads to cellular death. da Silva Santos et al. [12] study liver alterations in mice treated with UA and also showed morphological changes (hepatic cell plates derangement and inflammatory infiltrate). In this study, UA encapsulation into PLGA (poly(lactic-co-glycolic acid)) nanocapsules decreased the hepatotoxicity in vivo. To the best of our knowledge, studies related to histopathological alterations in the renal morphology of animals treated with UA have not been established to date. Kidney tissue sections of UA animals showed prominent renal damage including intense nuclear acidophilia and cytoplasmic vacuolation, capsular space and convoluted tubules lumen size increase, fluid accumulation between the basal membrane and cuboidal epithelial cells of the convoluted tubes, and cytoplasmic material accumulation in the renal tubules and cell lysis. On the other hand, the UA-micro-treated group (2000 mg/kg; p.o.) showed slight derangement of the simple cuboidal epithelium of convoluted tubules and vacuolation presence, while renal corpuscles were kept preserved. The mechanisms that underlie kidney injury following UA treatment remain unclear. Further insight into these histological considerations needs further investigation.

The results in the present study indicated that free UA and UA-micro attenuated the carrageenan-induced rat paw edema and the levels of MPO (one inflammatory signal secreted after polymorphonuclear leukocytes are stimulated) into injured paw. Neutrophil granules myeloperoxidase (MPO) is involved in reactive oxygen production, free radicals, and membrane oxidation, and tissue MPO levels become higher during inflammatory conditions, which are commonly related with bacterial lysis and tissue oxidative injury [33]. Inflammatory cells, such as neutrophils and macrophages, play an important role in the inflammatory process through proinflammatory mediator secretion, including NO, TNF-*α*, and IL-1*β* [34]. In addition, a decrease in the leukocytes migration and TNF-*α*, IL-1*β*, and NO levels in the inflammatory exudate from subcutaneous air pouch was also registered. TNF-*α* and IL-1*β* are related to increase of vascular permeability and migration of cells that produce chemokines [35]. The results of our study corroborate with those developed by Vijayakumar et al. [23], which showed anti-inflammatory activity of UA in carrageenan-induced rat paw edema, and Huang et al. [6], which demonstrated that the anti-inflammatory effect of UA is related to the negative regulation of iNOS, IL-1*β*, and TNF-*α*. Overall, the results of the current study indicate that equivalent or greater anti-inflammatory activity was observed as UA was incorporated into poly-*ε*-caprolactone microsphere polymers (UA-micro).

## 5. Conclusion

UA is now shown to achieve equivalent or greater anti-inflammatory activity as it is incorporated into poly-*ε*-caprolactone microsphere polymers (UA-micro) compared to free UA, with greatly reduced acute toxicity.

## Figures and Tables

**Table 1:** Encapsulation efficiency, mean particle size (*d*_*v*_), polydispersion *(span)*, and zeta potential (*ζ*) of PCL microspheres with (UA-micro) and without usnic acid (PCL microspheres). Results were expressed as mean ± SD.

Drug: polymer	Encapsulation efficiency ± DP^*∗*^ (%)	*d*_*v*_ (*µ*m)	*Span*	Zeta potential (*ζ*) ± DP^*∗*^ (mV)
PCL microspheres	—	9.37	2.18	−26.9 ± 0.58
UA-micro	97.72 ± 0,00	13.54	2.36	−44.5 ± 2.95

^*∗*^SD: standard deviation.

**Table 2:** Usnic acid (UA) and encapsulated usnic acid (UA-micro) effects over Swiss mice physiological parameters (*n* = 6 animals/group).

Groups	Dose/p.o. mg/kg	Number of deaths	Food consumption (g/day)	Water consumption (mL/day)	Body weight (g)	
					Early	End
Control		0/6	25.11 ± 2.3	45.8 ± 1.3	32.6 ± 2.77	33.0 ± 3.2
UA-micro	2000	0/6	25.52 ± 2.2	46.4 ± 2.1	30.3 ± 3.02	32.3 ± 1.2
UA	2000	6/6	—	—	29.7 ± 1.34	—
UA	300	0/6	20.61 ± 2.4^*∗∗∗*^	44.4 ± 1.2	28.9 ± 1.27	30.3 ± 1.0^*∗∗*^
UA	50	0/6	23.75 ± 2.5	46.6 ± 3.1	30.6 ± 0.34	32.8 ± 1.84

^*∗∗*^ *p* < 0.01 and ^*∗∗∗*^*p* < 0.0001 when compared with control group.

**Table 3:** Anti-inflammatory effect of free usnic acid (UA), encapsulated usnic acid microspheres (UA-micro), and indomethacin on carrageenan induced rat paw edema model (*n* = 5 animals/group).

Groups	Variation of paw volume (mL)			
	60 min	120 min	180 min	240 min
Vehicle (control)	0.61 ± 0.05	1.25 ± 0.09	1.62 ± 0.09	1.30 ± 0.07
UA 25 mg/kg	0.32 ± 0.02^*∗*^	0.50 ± 0.04^*∗*^	1.10 ± 0.11^*∗*^	0.81 ± 0.14^*∗*^
UA-micro 25 mg/kg	0.30 ± 0.03^*∗*^	0.45 ± 0.05^*∗*^	0.87 ± 0.06^*∗*#^	0.68 ± 0.05^*∗*#^
UA-micro 50 mg/kg	0.26 ± 0.01^*∗*^	0.40 ± 0.03^*∗*^	0.63 ± 0.03^*∗*#^	0.48 ± 0.03^*∗*#^
Indomethacin 10 mg/kg	0.18 ± 0.02^*∗*^	0.30 ± 0.03^*∗*^	0.40 ± 0.03^*∗*^	0.29 ± 0.02^*∗*^

Values are expressed as mean ± SD (*n* = 5/group). ^*∗*^Significantly different from the control group, *p* < 0.05 (control versus UA 25 mg/kg; UA-micro 25 mg/kg; and UA-micro 50 mg/kg). ^#^Significantly different from the UA 25 mg/kg group, *p* < 0.05 (UA 25 mg/kg versus UA-micro 25 mg/kg and UA-micro 50 mg/kg).

**Table 4:** Effect of free usnic acid (UA) and encapsulated usnic acid microspheres (UA-micro) on neutrophils migration.

Groups	Dose (mg/kg p.o.)	Leukocytes migration (10^3^/mm^3^)	Inhibition%
Control	—	11.4 ± 1.17	—
UA	25	5.8 ± 0.68^*∗*^	49
UA-micro	25	7.8 ± 0.67^*∗*^	31
UA-micro	50	3.8 ± 0.49^*∗*#^	67
Indomethacin	10	3.6 ± 0.76^*∗*^	68

Data are shown as mean ± SD (*n* = 5/group) and the statistical significance between groups was determined using Analysis of Variance (ANOVA) followed by Turkey's test. ^*∗*^*p* < 0,0001 versus control. ^#^Nonsignificant versus indomethacin.
